# Proteomic and Transcriptomic Techniques to Decipher the Molecular Evolution of Venoms

**DOI:** 10.3390/toxins13020154

**Published:** 2021-02-16

**Authors:** Stephanie Mouchbahani-Constance, Reza Sharif-Naeini

**Affiliations:** Department of Physiology and Cell Information Systems Group, Alan Edwards Center for Research on Pain, McGill University, Montreal, QC H3A 0G4, Canada; stephanie.mouchbahaniconstance@mail.mcgill.ca

**Keywords:** venom, toxin, predator, prey, high-performance liquid chromatography, mass spectrometry

## Abstract

Nature’s library of venoms is a vast and untapped resource that has the potential of becoming the source of a wide variety of new drugs and therapeutics. The discovery of these valuable molecules, hidden in diverse collections of different venoms, requires highly specific genetic and proteomic sequencing techniques. These have been used to sequence a variety of venom glands from species ranging from snakes to scorpions, and some marine species. In addition to identifying toxin sequences, these techniques have paved the way for identifying various novel evolutionary links between species that were previously thought to be unrelated. Furthermore, proteomics-based techniques have allowed researchers to discover how specific toxins have evolved within related species, and in the context of environmental pressures. These techniques allow groups to discover novel proteins, identify mutations of interest, and discover new ways to modify toxins for biomimetic purposes and for the development of new therapeutics.

## 1. Introduction

Our planet possesses libraries of molecules that have the potential to become the next generation of therapeutics, and these libraries are located within venoms. Venoms are secretions produced by animals and are composed of a cocktail of toxin molecules that work together to execute the venom’s final function. Venoms give species an adapted advantage for either defense, predation, or competition, but understanding exactly what these adaptations are and how they came about mostly remains a mystery [1,2]. Venomics describes the integrated study of venoms from a genomic, transcriptomic, and proteomic point of view, to uncover the molecular underpinnings of venoms and the glands that produce them [3]. Furthermore, by studying evolutionary adaptations of venoms to different environments and other evolutionary pressures, we can understand how certain evolutionary toxin modifications may ascribe an advantage to a given molecule. These modifications can then be harnessed and applied to drug design processes, allowing us to develop more efficient, effective, and stable drugs [4].

Both predator and prey species have evolved venoms—in the case of the predator as an aid in catching dinner, and in the case of prey as a protective mechanism against becoming dinner. The evolution of these tools, and various resistance mechanisms against them, has resulted in an evolutionary arms race. If a predator happens to lose the race, it can still hunt again, but if the prey loses, it will die. Thus, adaptation plays a central role in driving the phenotypic evolution of venoms. By unveiling the genetic variants that underlie these changes, one can begin to uncover the molecular basis of different evolutionary processes as they relate to venoms [5,6,7,8].

From a predator’s point of view, venom is its most important weapon for capturing prey. To maximize the chances of a meal, these venoms have evolved an impressive diversity of biochemical components. In addition to a powerful chemical weapon, many species have also optimized their venom administration techniques (such as the miniature harpoon-like teeth from cone snails, which serve to directly inject venom into victims [9]) to guarantee the incapacitation of their prey [10]. For many species, these venom delivery systems can be observed by the naked eye, but the complex composition of the key weapon—the venom—remains to be uncovered. These venoms are almost analogous to a molecular swiss-army knife, with each “arm” being a toxin that has been evolutionarily specialized to execute its particular role.

This review will focus on overviewing the current techniques in venom proteomics, as well as venom gland transcriptome sequencing. We will further describe how advances in these techniques have allowed the field to slowly uncover the molecular composition of nature’s vast microscopic arsenal of chemical weapons: venoms. Furthermore, we will address how future developments in this field will provide insights into the molecular evolution of venoms, which are key cheat codes that we can apply to any molecule in the future to maximize its efficiency.

## 2. Techniques in Venomics

**Figure 1:**
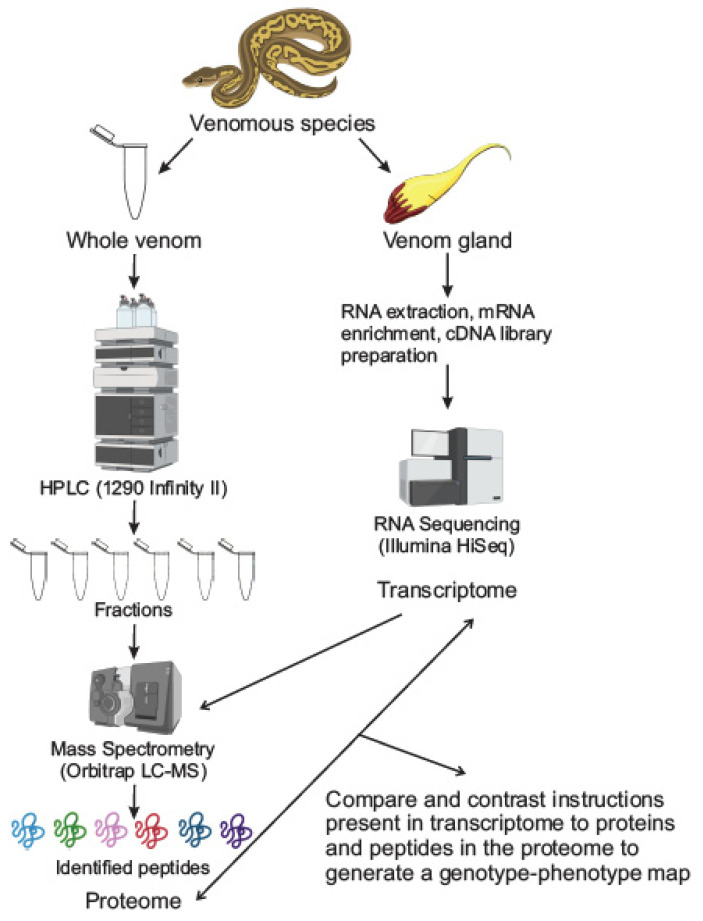
Classic workflow for studying toxin components from venoms. In most studies, the series of experiments employed to elucidate the molecular components of venoms involve studying the venom gland and the venom itself separately. The venom gland is subject to RNA extraction, mRNA enrichment, cDNA library preparation and RNA sequencing to obtain the gland’s transcriptome. In parallel, the whole venom is fractionated using any number of separation techniques, high-performance liquid chromatography (HPLC) is pictured above. These fractions are subject to mass spectrometry to identify peptides and obtain a proteome with the gland’s transcriptome (obtained from RNA sequencing) being utilized as a reference database for the mass spectrometry.

Historically, a generally similar workflow has been followed for elucidating the molecular components of venoms (see Figure 1). Typically, following venom extraction and purification, groups follow separation techniques to separate the venom into specific fractions based on one, or a combination of different molecular properties ranging from size, polarity, and charge to solubility and substrate affinity. One such example of a fractionation technique is high-performance liquid chromatography (HPLC). Groups would then inject fractions into mice to observe which fraction elicited the final behavior of interest. Finally, “active” fractions were purified to homogeneity with subsequent fractionations, and then amino acid sequencing would be determined by reducing the toxins and carboxy-methylating them. These could then be analyzed using Edman degradation with devices, such as the Procise sequencing system or the Beckman890D spinning-cup sequencer [10,11]. However, these techniques were complicated to execute, and additional levels of complexity were added by the fact that toxins are often multi-peptide proteins, which could not be identified using such basic techniques.

### 2.1. Proteomics: Separation and Mass Spectrometry Techniques

#### 2.1.1. General Overview of Separating Proteins, Bottom up vs. Top down

It is clear that the most efficient approach to increasing the resolution of current sequencing techniques lies in the proteomic steps that precede them. By using the modern methods described herein, it is possible to achieve a high efficiency of toxin fractionation and sequence identification to reliably characterize venoms. These methods improve the fractionation and separation of toxin isoforms, allowing for sequencing techniques to analyze a higher diversity of toxin isoforms, as opposed to older techniques that may not separate highly similar toxin fragments as efficiently. This increased resolution at the protein level can provide greater insights into toxin variability among highly similar isoforms and identifies different single nucleotide polymorphisms (SNPs) and post-translational modifications (PTMs) that may occur within toxin families. Ultimately, these techniques will provide the field with much deeper insights into toxin evolution [12].

**Figure 2:**
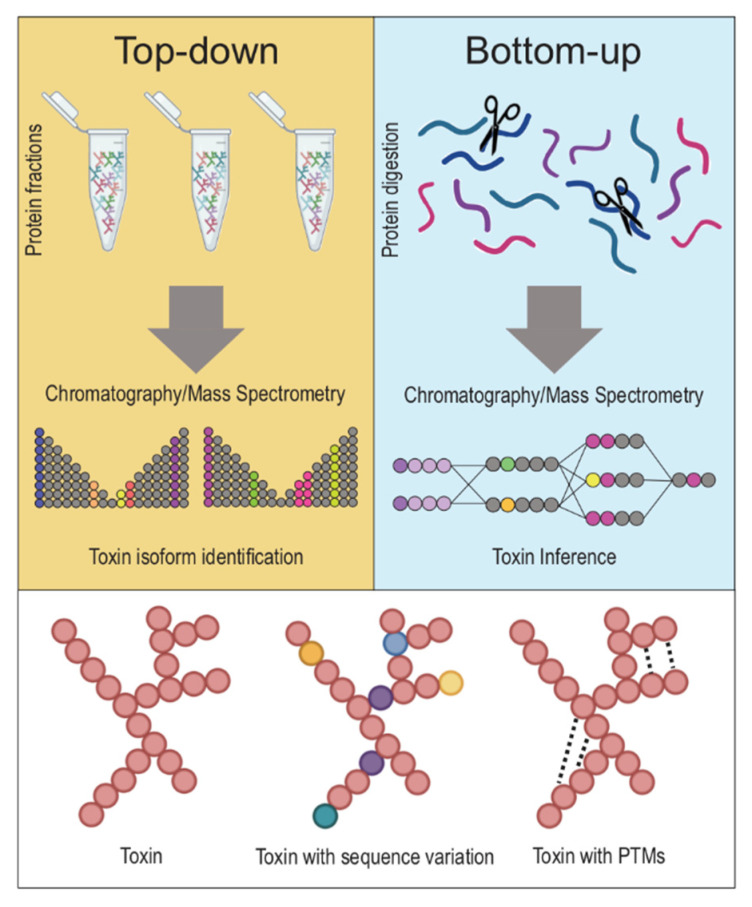
Top-down vs. Bottom-up proteomics approaches. The two main approaches in discerning toxins from one another in venoms are top-down and bottom-up proteomics. Top-down proteomics describes an approach whereby a venom’s whole proteins (notice: native, folded proteins in tubes on top left) are holistically analyzed without the need for breaking proteins down into their constituent fragments. On the other hand, bottom-up proteomics refers to techniques that involve the denaturing of whole proteins (notice: unfolded proteins on top right) into fractions, and the study of these protein fractions separately, before reassembling these fragments into proteins in silico to identify a venom’s constituent toxin proteins. Typically, top-down proteomics is more accurate in discerning between closely related toxins with minimal sequence variation or toxins with post-translational modifications.

Historically, toxin elucidation has occurred mostly using bottom-up proteomic (BUP) studies (see Figure 2), whereby toxin proteins are broken down into peptides using enzymatic or chemical reactions, then fractionated, typically with liquid chromatography (LC)-based technique and then identified by studying their intact mass and fragmentation patterns and comparing them to a protein sequence database [12,13,14]. This approach has been used in toxin studies, since early gel-based proteomics until shotgun proteomic techniques [13]. However, peptide-centric proteomics cannot necessarily give an accurate biological interpretation of toxins, since different peptides may be present in different combinations in multiple toxins, may be present in different proteoforms, or may have undergone post-translational modifications with major functional implications, termed the “protein inference problem” [14].

With technological developments, new methods have been developed which allow for top-down proteomic (TDP) studies. In these, rather than breaking proteins down into their component peptides, intact proteins (or gas-phase fragmented proteins) can be holistically analyzed using tandem mass spectrometry techniques without any requirement for digestion, although some degree of denaturing can also be done to compare the resulting proteomes of denatured vs. native samples (see Figure 2) [15,16,17]. The fragmentation patterns of these whole proteins can bring to light information about toxins that would otherwise be inaccessible using traditional bottom-up techniques, such as identification of proteoforms, post-translational modifications (PTM), and alternative splicing [12,15,16,17,18,19,20,21,22,23].

Top-down proteomics allows the field to study how changes to toxins may ascribe evolutionary advantages to venomous species, a possibility that was unlikely by simply studying a venom gland’s transcriptome. This is especially advantageous for investigating the venoms of novel subspecies, whose close relatives have already been studied using a bottom-up approach [19]. For these, TDP can not only save time in analyzing toxin components, but also provide new insights into the specific evolution of that species in comparison to its close relatives, uncovering the unique traits of this subspecies.

Furthermore, TDP will allow for in-depth investigations of intraspecies venom variations, and may bring to light environmental factors that influence a venom’s composition, such as diet, which is an influencing factor in the venoms of certain species of snakes and tetrapods [6,24,25,26,27,28,29,30,31].

#### 2.1.2. Separation Techniques

**Figure 3:**
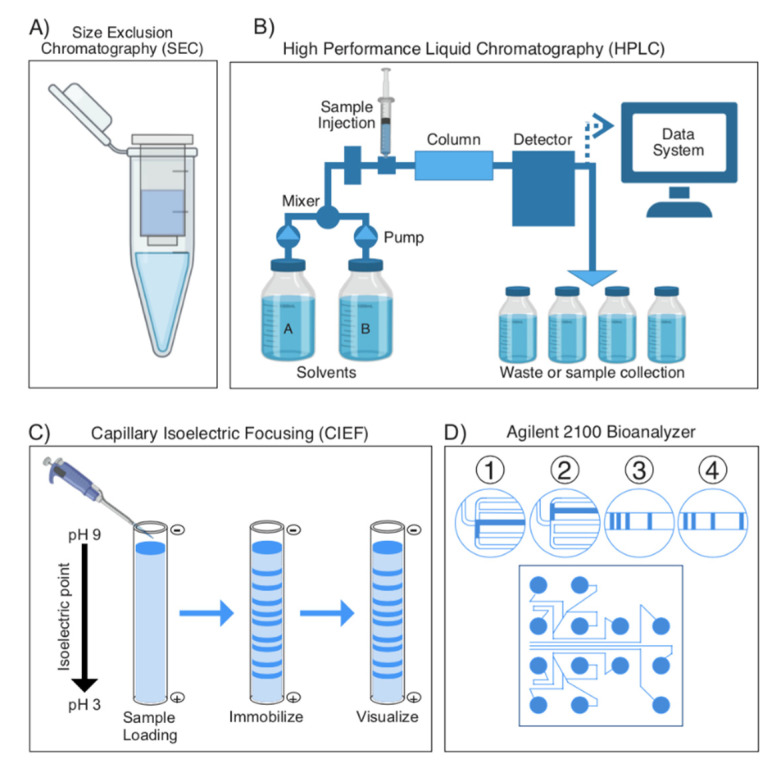
Overview of most commonly used sample separation techniques in venomics. (**A**) A molecular weight sieve is used for size exclusion chromatography to separate samples based on their size or molecular weight. (**B**) High-performance liquid chromatography (HPLC) is used to separate analytes in a sample based on their polarity. Analytes interact with a column (stationary phase) differently based on their polarity, causing them to elute at different speeds. The eluted molecules are detected by either a spectroscopic or an electrochemical detector, with a readout available to the experimenter describing the eluate’s absorbance at different time points. (**C**) Capillary isoelectric focusing (CIEF) is a separation technique that separates molecules based on their isoelectric points in fused silica capillary tubes. (**D**) The Agilent 2100 Bioanalyzer is an innovative new “venom-on-a-chip” technology that incorporates a variation of CIEF in which microchannels host an electrophoretic separation of proteins, which are then detected via fluorescence. The software then transforms this data into gel-like images and electropherograms for easy interpretation.

Most venoms are complex mixtures of proteins, making fractionation a necessary task as mass spectrometry (MS) acquisition technologies can often not handle such diversity [12]. These fractionation techniques are numerous, and some of the most common, to be reviewed below in more detail, are: Reversed-phase high-performance liquid chromatography (RP-HPLC, otherwise known as HPLC) (Figure 3B), capillary isoelectric focusing (CIEF) (Figure 3C), size-exclusion chromatography (SEC) (Figure 3A), capillary zone electrophoresis (CZE) (Figure 3D), and 2D-Gel Electrophoresis (2D-GE) [20,31,32,33,34,35,36,37,38,39].

In HPLC, the sample is prepared in a solvent, a polar mobile phase, and is pumped through a column, a nonpolar stationary phase, whose size and packing material may vary. Based on the polarity of the sample’s components, they will each interact differently with the stationary phase: Those that are most polar will interact the least with the stationary phase, and thus, elute first, with the opposite being true for the sample’s most nonpolar components. These separated components can be detected with either spectroscopic detectors or electrochemical detectors to determine both elution time, as well as the component’s relative concentration in the sample [40]. One of the most important advances in HPLC technology has come from adopting small particles (<2 μm) in the column’s packing material. Smaller particle size results in the narrowing of chromatographic peaks without varying the center-to-center distance between peaks, ultimately improving a column’s resolution and sensitivity [32].

Furthermore, varying the polarity of the stationary phase and varying the column’s length or the pump’s flow rate, can all vary the speed of sample elution and ultimately affect the sample’s retention time in the stationary phase. The slower flow rate allows for a better separation of the sample’s components, especially if it is a medley of molecules as with venoms [41,42], but this comes at the expense of reduced separation resolution. Lower flow rates cause analytes to increasingly diffuse longitudinally within the column, which ultimately results in a less precise separation of these analytes based on their polarity. Spectroscopic detection of these samples will typically show broader and muddled peaks, leading to a lower resolution sample separation.

CIEF is based on the technique of capillary gel electrophoresis (CGE), in which molecules are separated in a gel, based on their different isoelectric points (Figure 3C). In CIEF, however, instead of using gels, the separation is performed in fused silica capillary tubes (internal diameter of 25–100 μm). Within the capillary, proteins migrate in response to an electric field based on their isoelectric point and become focused at a point where their net charge is balanced. Focused zones are then transported past a monitoring point to detect the now separated proteins. By using a fused silica capillary tube, heat is efficiently diffused, allowing higher voltages to separate a broader range of proteins. This is essential, as introducing excessively high temperatures may introduce extraneous protein denaturing [42,43,44].

CZE is another separation technique where sample components are separated based on their charge. In this technique, a capillary column is immersed in two buffer-filled reservoirs, to which a high voltage is applied. The sample is then injected into the capillary, and its components are separated based on both the electrophoretic forces, as well as the developing electro-osmotic forces within the capillary. This technique provides a higher resolution, in terms of component separation, compared to HPLC, since peaks tend to be very narrow. However, since flow rates tend to be low (nL per minute range), due to a very small amount of starting material, CZE must be directly coupled with mass spectrometry cannot be coupled with another downstream fractionation technique. To avoid potential loss of material between fractionation and analytical steps, CZE is often directly interfaced with nanospray MS, making it impossible to couple with another downstream fractionation technique before MS. Due to this, CZE is not a reliable method for quantifying a given component in a sample [45,46].

SEC is a chromatographic method in which hydrophilic molecules (proteins or other solutes) are separated based on their size or molecular weight, either through molecular-weight sieves or through gel-filtration chromatography [47]. It has proven to be very useful in separating proteins, due to the technique’s ability to separate native proteins (undigested) or protein complexes. This is essential in the field of venomics, as toxins are often large proteins that may or may not exist in complexes with other proteins [48,49]. This technique can also be achieved using HPLC to perform a high-resolution fractionation of molecules based on their molecular weight [47]. However, depending on the separation technology used to achieve SEC, it is not a particularly high-resolution chromatographic technique and has a limited dynamic range for protein separation. For example, the use of molecular-weight sieves, while very user-friendly, has a far lower chromatographic resolution than HPLC would [47].

2-dimensional gel electrophoresis (2DGE) is another common and highly accessible method for groups to separate proteins in venoms and in order to characterize these venoms. 2DGE separates proteins according to two different variables in the same gel. In one dimension, proteins can be separated based on their isoelectric point, and in the other by their relative molecular weight [50,51]. Based on the gel’s composition and buffers used, 2DGE has the potential to resolve up to 10,000 proteins in a single gel, which can be analyzed after capturing an image of the gel. The technique has its limitations: It cannot be used to analyze a venom’s entire proteome as it has difficulty resolving a sample’s smallest and largest proteins, as well as proteins that are highly acidic and highly basic. That being said, 2DGE can separate and display up to thousands of proteins in one single lane of a gel and ultimately enabling the visualization of numerous protein isoforms [51,52,53,54]. Furthermore, gel electrophoresis can be carried out in native or denaturing conditions depending on the gel used. To carry out electrophoresis of non-denatured (intact) proteins and protein complexes, one can use techniques such as Blue Native-PAGE (BN-PAGE) or Clear Native electrophoresis to separation and isolation of protein complexes (of particular interest in venom studies) [55,56]. With denaturing conditions, such as SDS-PAGE, one can instead separate and isolate denature proteins, in which secondary structures have been broken down. However, it should be noted that denaturing conditions tend to be preferable when the experimental intention is to isolate proteins for protein sequencing [57]. 

A highly innovative hardware/software combination that automates the detection and separation of proteins in venom is the Agilent 2100 Bioanalyzer, which enables rapid, semi-automated “venom-on-a-chip” proteomic analyses. On a single chip, this technology uses a variation of CIEF (Figure 3D). It uses microchannels that are etched into the chip, allowing for the electrophoretic separation of proteins, and then detection via fluorescence. The software transforms this data into gel-like images and electropherograms for easy interpretation, allowing for complete separation and detection of protein components with minimal effort from researchers [58,59,60,61,62]. Side-by-side comparisons of this technique with pre-fractionated samples, and comparisons with other techniques, shows that it is highly efficient at discriminating interspecies, as well as intraspecies venom variations [58]. Nonetheless, it is important to note that while this technique is very useful from an electrophoretic point of view, it is currently impossible for a Bioanalyzer to return a sample after separation. This makes the technique impossible to interface with MS to subsequently identify separated peptides. Despite this, as the field of venomics is rapidly growing, technologies, such as the Agilent 2100 Bioanalyzer will make venomic studies more accessible to a broader range of laboratories, and will allow for simultaneous, high-throughput studies of venoms from a variety of species and from multiple individuals.

#### 2.1.3. Mass Spectrometry Techniques

**Figure 4:**
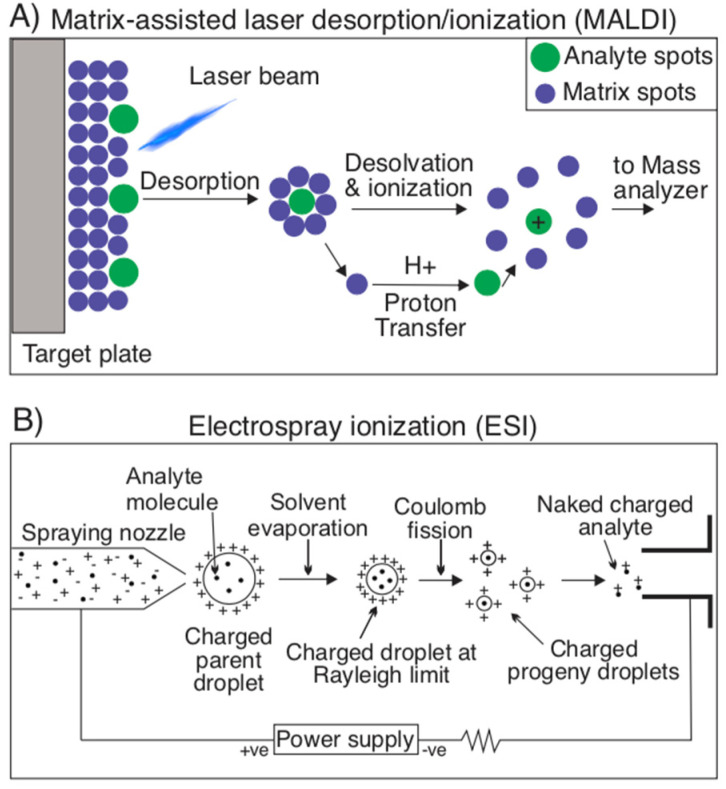
Ionization techniques for mass spectrometry. (**A**) Matrix-assisted laser desorption/ionization (MALDI) is an ionization technique used for mass spectrometry that involves sublimating an analyte molecule embedded in a matrix of small molecules. During this sublimation, the analyte itself is not fragmented, but rather ionized thanks to the protonating/deprotonating properties of the matrix molecules. (**B**) Electrospray ionization (ESI) is another ionization technique that consists of the use of high voltage and a nebulizing gas to vaporize a solvent, containing analytes, into an aerosol. The remaining solvent in these droplets is evaporated, leaving a charged droplet, which undergoes Coulomb fission resulting in charged progeny droplets. From these droplets, naked charged analytes are detected by the device’s detector to investigate their *m*/*z* ratio.

Modern proteomics has embraced mass spectrometry to identify proteins based on an analysis of the mass/charge ratio obtained from ionizing such proteins or peptides. In order to perform an analysis of mass/charge ratio (*m*/*z*), proteins must be ionized. For this, two techniques prevail as most efficient at preventing proteins from fragmenting in the ionization process: Matrix-assisted laser desorption ionization (MALDI) and Electrospray Ionization (ESI). In MALDI, a laser strikes a matrix of small molecules, in which an analyte is embedded, sublimating the analyte molecule without fragmentation and ionizing it, since the small matrix molecules can either protonate or deprotonate it (Figure 4A) [20,50,63,64,65]. In ESI, a high voltage, coupled with a parallel flow of nebulizing gas (usually nitrogen) is used to vaporize a liquid solvent to create an aerosol. This solvent varies based on the sample to be analyzed, but is typically composed of a polar solvent (for example, H_2_O, acetonitrile, tetrahydrofluoran) (Figure 4B). It is most unique from MALDI in that it is better at preventing molecule fragmentation and can produce multiple-charged ions, which can extend the ionizer’s range to accommodate very large molecules in the kDa to MDa range [65,66,67].

**Figure 5:**
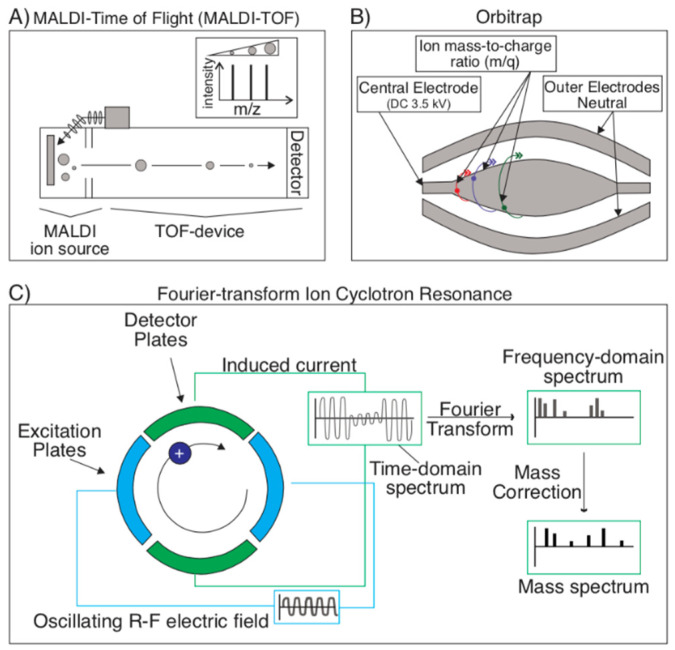
Detection techniques for mass spectrometry. (**A**) Time-of-flight (TOF) is the most common detection technique in mass spectrometry and is often coupled with MALDI. Ionized analyte particles travel at rates proportional to their mass. TOF utilizes this variable to infer particle size and (along with the reference database and various software) composition. (**B**) Orbitraps utilize ion traps to identify an analyte’s mass. These ion traps consist of trapping ionized particles between an outer electrode and inner electrode and performing a Fourier transform on the charge frequency pattern to produce the ion’s mass spectrum. (**C**) In a Fourier-transform ion cyclotron resonance (FT-ICR) device, ions are instead trapped in a Penning trap (which uses a magnetic field to trap ions radially and an electric field to confine particles axially). Ions will rotate at their preferred frequency in packets, which produce a free induction decay (FID) charge as they pass a pair of electrodes. This FID is a time-domain spectrum, from which a frequency-domain spectrum can be extracted via a Fourier transform. Following a mass correction, the sample’s mass spectrum can be produced from the frequency-domain spectrum.

Following ionization, the ions must be detected to determine the *m*/*z*. Currently, the most common MS detection technique is time-of-flight (TOF), often coupled with MALDI (Figure 5A). The main principle of TOF is that ions, in-flight following ionization, travel at a rate proportional to their mass: Heavy ions take longer to reach the detector than lighter ones, since they all begin their journey to the detector at the same time and place [20,50,63,64,65]. However, in situations where MALDI is not the optimal ionization technique (i.e., for the detection of larger peptides or proteins), ESI may also be interfaced with a TOF analyzer (ESI-MS/MS). This can be done by coupling the ESI with quadrupole MS to act as a mass filter, followed by an orthogonally placed TOF analyzer using a reflectron to reflect the ion beam towards the TOF detector [68]. ESI may also be coupled with an Orbitrap or a Q-Orbitrap to increase resolving power over an ESI-TOF system [69].

Other detection techniques utilize frequency rather than time as a measure of an ion’s mass; these include Orbitrap (Figure 5B) and FT-ICR (Fourier transform ion cyclotron resonance) (Figure 5C). With more resolving power than TOF, the Orbitrap utilizes an ion trap to analyze mass by trapping ions between an outer barrel-shaped electrode and an inner rod-shaped electrode. Ions orbit around the inner electrode, and a Fourier transform of the resulting charge frequency pattern reveals the ion’s mass spectrum [70,71]. In FT-ICR, ions are instead trapped in a Penning trap (which uses a magnetic field to trap ions radially and an electric field to confine particles axially), where they’re excited at their preferred cyclotron frequency to a larger cyclotron radius by the axial oscillating electric field. Ions rotate at their preferred cyclotron frequency in packets, producing a free induction decay (FID) charge when they pass a pair of electrodes, which is essentially a superposition of sine waves. Using a Fourier transform, the mass spectrum can be extracted from this data sinusoidal FID charge data [63,72,73,74]. Since FT-ICR uses a superconducting magnet instead of radio-frequency voltage, it is a much more stable device that can ultimately read more accurate masses at a higher resolution.

### 2.2. Transcriptomics

#### 2.2.1. Transcriptomics: Sequencing Techniques

Early on, polymerase chain reaction (PCR) allowed for the study of different components of the venom gland at the transcriptome level, with cDNA libraries identifies the numerous venom components. However, the real game-changer came when high-throughput sequencing technologies came along, such as Next Generation Sequencing, and all the platforms that allow for it (pyrosequencing, Illumina, SOLiD, ion semiconductor, DNA nanoball, etc.). All of these have proven to be of the utmost importance in the generation of de novo venom gland transcriptomes [75].

Most RNA-sequencing based assays begin with similar early-stage workflows: RNA is extracted, ribosomal RNA is depleted, or mRNA is enriched, cDNA is synthesized, and an adaptor-ligated sequencing library is prepared. Depending on the high-throughput sequencing platform utilized, the library is sequenced to a read depth of 20+ million reads per sample. The number of reads per sample may change from one sequencing platform to another, and it is a crucial variable in the quantitative capacity of an RNA-sequencing experiment. As the number of reads per sample increases, more transcripts can be detected, increasing the sample’s sequencing depth and ultimately quantifying these transcripts to be more precise [76]. Finally, the sequencing reads are aligned and assembled into the final transcriptome utilizing computational steps [77,78]. These data can be analyzed for a variety of purposes, with over 100 different RNA-Seq methods that have been derived from the standard protocol [77].

Typically, most venom gland transcriptomes that have been sequenced to date have been read using short-read sequencing technology, often Illumina sequencing, in part due to its relatively low cost and easy to implement, but primarily due to the high quality, quantitative data that can be obtained using this technique [79,80,81,82,83,84,85,86,87,88,89,90,91,92,93]. Generally, sequenced cDNA fragments are under 200 bp in length with 20–30 million reads per sample, depending on the technology and experimental limitations. This technique is very robust, and has been verified using large-scale comparisons of short-read sequencing data, which showed high intra-platform and inter-platform correlations [94]. However, multiple gene isoforms pose an issue in transcriptome-wide analyses using short-read RNA-seq, since transcript isoforms may be longer than 200 bp (for example, 50% of transcripts are longer than 2500 bp in the human genome [95]). A single venom can contain multiple isoforms of a single toxin, an important point to consider when performing short-read sequencing on venom glands, especially when the source of such isoform variation is not yet known (PTMs, SNPs, etc.).

While short-read RNA-seq can be achieved using Illumina technology, long-read RNA-seq can be achieved using platforms, such as those from Pacific Biosciences and Oxford Nanopore. These techniques can allow for sequencing of an entire, complete RNA molecule following reverse transcription, circumventing the issues posed by short-read sequencing techniques and reducing sequencing ambiguities of potential toxin isoforms in a given sample [77,78,96,97,98]. However, long-read sequencing technologies are limited in the number of reads they can perform per samples: While short-read sequencing allows for 20–30 million reads per sample, long-read sequencing techniques are relatively low-throughput and only allow for 500,000–10 million reads per sample, thus potentially reducing the quantitative capacity of this technique and ultimately making it less valuable for differential gene expression analyses. However, its ability to differentiate between isoforms makes it ideal for de novo transcriptome assembly [77].

#### 2.2.2. Current Gold Standards in Transcriptomics

The current gold standard for whole venom gland RNA-seq has been Illumina HiSeq sequencing, using Trinity software to generate a de novo assembly and Trinotate software to annotate the assembly [1,2,3,99,100,101]. This will certainly change as long-read RNA-seq techniques are refined and upgraded. Improved resolution of proteomic techniques has started to demonstrate the wide variety of toxin isoforms in a given venom sample, and transcriptome sequencing can provide insights into how these isoforms evolved from one another, thus offering a unique view into the evolution of prey and predators. Gene duplication and isoform evolution are common events in the evolution of toxin isoforms—to gain an understanding of how toxin isoforms go through this evolution, it is essential for us to gain insights into what modifications provide toxins with a selective advantage [102,103,104,105]. As mentioned above, short-read RNA-seq techniques are limited by read length, which has been improved upon in the relatively young long-read techniques. As these long-read techniques are upgraded, we will certainly be able to gain much higher resolution, quantitative reads of long-read sequenced transcriptomes, ultimately providing us with transcriptome-level insights into toxin isoform evolution.

### 2.3. Integrated Proteomic-Transcriptomic Techniques

The proteomic and transcriptomic techniques described in this review are powerful tools in analyzing venoms and elucidation of individual toxins. However, integrating proteomic and transcriptomic techniques in analyzing venoms presents an even stronger approach that can allow for a rapid and thorough analysis of venom components, and has become very common in the field of venomics [5,93,106,107]. While transcriptomics provides a relatively unbiased representation of the diversity of transcripts in a given sample, the technique also captures thousands of non-venom peptide encoding transcripts (which may be part of other cellular processes) and cannot detect post-translational modifications, which have important consequences for a toxin peptide’s functionality [108]. The combination of transcriptomics and proteomics allows for the transcriptomics arm of experimentation to capture a sample’s high transcript diversity, and the proteomic arm allows for the narrowing of this diversity to focus analyses only on toxin peptide-encoding transcripts. Furthermore, following identification of toxin-encoding transcripts, many transcriptomic techniques can provide insights into the relative expression level of these transcripts, giving expression estimates for toxin-related genes of interest [108,109,110].

## 3. Conclusions

The sequencing of venom glands and the parallel sequencing of these venoms’ target species, has allowed science to write out a so-called “molecular storyline”, hinting at how much resistance mechanisms have evolved, and how venoms themselves have evolved in response to maintain their evolutionary arms race [111,112,113,114,115,116,117]. For many years, our understanding of the basic principles of evolution has relied on the idea that adaptations to change are necessary for survival. An increasing number of discoveries in the study of venoms and the evolution of their targets seem to confirm that one of the building blocks of survival throughout evolution is indeed a species’ genomic thriftiness. Nature has endowed species with the possibility for genomic plasticity in order to survive the constant arms race of predator-prey interactions [118,119].

By gaining an understanding of how and why certain genes become plastic in the evolution of venoms and their resistance mechanisms, we can begin to decipher nature’s evolutionary methods and can start to apply these learned concepts to the treatment of diseases.

## Data Availability

Not applicable.
